# Serum lysophospholipid levels are altered in dyslipidemic hamsters

**DOI:** 10.1038/s41598-017-10651-0

**Published:** 2017-09-05

**Authors:** Susana Suárez-García, Antoni Caimari, Josep Maria del Bas, Manuel Suárez, Lluís Arola

**Affiliations:** 1 Nutrigenomics Research Group, Department of Biochemistry and Biotechnology, Universitat Rovira i Virgili (URV), Tarragona, 43007 Spain; 2 Technological Unit of Nutrition and Health. EURECAT-Technological Center of Catalonia, Reus, 43204 Spain

## Abstract

Dyslipidemias are common disorders that predispose individuals to severe diseases. It is known that healthy living habits can prevent dyslipidemias if they are diagnosed properly. Therefore, biomarkers that assist in diagnosis are essential. The aim of this study was to identify biomarkers of dyslipidemia progression, which in turn disclose its etiology. These findings will pave the way for examinations of the regulatory mechanisms involved in dyslipidemias. Hamsters were fed either a normal-fat diet (NFD) or a high-fat diet. Some of the NFD-fed animals were further treated with the hyperlipidemic agent Poloxamer 407. Non-targeted metabolomics was used to investigate progressive changes in unknown serum metabolites. The hepatic expression of putative biomarker-related genes was also analyzed. The serum levels of lysophospholipids (Lyso-PLs) and their related enzymes lecithin-cholesterol acyltransferase (LCAT), secreted phospholipase A_2_ (sPLA_2_) and paraoxonase-1 were altered in dyslipidemic hamsters. Lysophosphatidylcholine levels were increased in diet-induced dyslipidemic groups, whereas lysophosphatidylethanolamine levels increased in response to the chemical treatment. The liver was significantly involved in regulating the levels of these molecules, based on the modified expression of endothelial lipase (*Lipg*), sPLA_2_ (*Pla2g2a*) and acyltransferases (*Lcat* and *Lpcat3*). We concluded that Lyso-PL evaluation could aid in the comprehensive diagnosis and management of lipid disorders.

## Introduction

Dyslipidemias are disturbances in lipid metabolism that have a high prevalence in both developed and developing countries and lead to changes in the levels and/or functions of the plasma lipoproteins^1–3^. These lipid disorders can appear as an elevation in the levels of circulating triglycerides, total cholesterol (TC) and low-density lipoprotein cholesterol (LDLc), as well as a reduction in high-density lipoprotein cholesterol (HDLc) levels^4^. Alterations in lipid metabolism and transport can have severe implications, predisposing the patients to the development of cardiovascular diseases, fatty liver disorders and different types of cancer^5–8^.

In terms of the etiology, the development of dyslipidemia in humans depends on both genetic and environmental factors. Thus, lipid management begins with the exclusion of secondary causes of metabolic disorder^9^. A diet rich in saturated fats and cholesterol is a well-established source of acquired dyslipidemias^4–6^, but accumulating evidence has shown that certain exogenous compounds, such as alcohol, tobacco or some drugs, also induce lipid disorders when consumed regularly over a long period of time^9–11^. Advances in our understanding of the mechanisms involved in dyslipidemia progression that depend on the type of triggering factor are necessary to improve the diagnosis and treatment of dyslipidemia-related diseases.

The Golden Syrian hamster is the rodent that displays greatest similarity to humans regarding lipoprotein metabolism^12^. In the present work, this hamster model was selected to investigate the biological alterations that occur after the chronic intake of a high-fat diet (HFD) for a maximum of 30 days. Furthermore, an additional group of animals was treated with the hyperlipidemic agent Poloxamer 407 (P407) to generate a different form of dyslipidemia that was not induced by diet. P407 is a hydrophilic, non-ionic, surface-active compound with low toxicity that has been extensively used in pharmaceutical preparations and in common personal care products, such as toothpastes and cosmetics^13^. After parental administration, P407 induces hypertriglyceridemia and hypercholesterolemia in rodents by inhibiting the heparin-releasable fraction of lipoprotein lipase (LPL) and the cholesterol 7α-hydroxylase (C7αH), respectively^14, 15^. However, chronic treatment with P407 does not seem to stimulate 3-hydroxy-3-methylglutaryl coenzyme A (HMG-CoA) reductase, a key enzyme involved in the synthesis of cholesterol, target of many anti-dyslipidemic drugs^16^. P407-induced hyperlipidemia, which exhibits more pronounced plasma triglyceride levels than cholesterol levels, is dose-dependent and remains at steady levels with repetitive injections of the drug approximately every 3 days^17^. Thus, the P407-treated rodent and the HFD-fed hamster are well-established models of atherogenesis that properly reproduce the adverse events that precede the development of atherosclerotic lesions in humans^18, 19^.

In this context, the use of an omics-based strategy allowed us to provide a global characterization of the changes in the circulating metabolome associated with the long-term administration of both pro-dyslipidemic treatments, as well as to investigate the main hepatic mechanisms. Some studies have been performed in HFD-fed hamsters^20–22^ and dyslipidemic subjects^23, 24^ using non-targeted metabolomics techniques, but, to date, research on P407-treated rodents has not utilized this approach. All these studies point to the importance of comprehensive investigations for expanding our understanding of the complex networks involved in the development of lipid disorders and the identification of novel biomarkers for the early diagnosis and management of related diseases.

Therefore, the main objective of the present study was to identify non-invasive biomarkers of dyslipidemia progression that reveal the etiology of the disorder. Therefore, we used ultra-high-performance liquid chromatography coupled to quadrupole time-of-flight mass spectrometry (UHPLC-Q-TOF-MS/MS) to evaluate the alterations in the serum metabolite levels of adult hamsters in response to different periods of HFD feeding and treatment with P407. Furthermore, after the untargeted identification of the metabolites associated with each type of dyslipidemia, the hepatic mechanisms involved in the metabolism of these molecules were also examined to extend our knowledge of the modes of action of the two treatments and their disparities.

## Material and Methods

### Chemicals

Methanol (Scharlab S.L., Barcelona, Spain) and glacial acetic acid (Panreac, Barcelona, Spain) were of high-performance liquid chromatography (HPLC) analytical grade. Ultrapure water was obtained from a Milli-Q advantage A10 system (Madrid, Spain). Phenylalanine^13^C (Fluka/Sigma-Aldrich, Madrid, Spain) was used as an internal standard (IS) for the untargeted metabolomics analysis^25^. The standard was dissolved in methanol at 1 mg/mL and stored at −20 °C prior to use. Butylated hydroxytoluene (BHT, Fluka/Sigma-Aldrich, Madrid, Spain) was added to the extraction solution to avoid metabolite oxidation during the sample extraction. P407 (Fluka/Sigma-Aldrich, Madrid, Spain) was administered to the animals to induce dyslipidemia.

### Animal studies

Two different *in vivo* studies were performed to identify serum biomarkers of dyslipidemia. All of procedures were approved by the Animal Ethics Committee of the University Rovira i Virgili (Tarragona, Spain) and they have been performed in accordance with the European Communities Council Directive (86/609/EEC).

A first exploratory study was performed using 3-month-old male Golden Syrian hamsters (Charles River Laboratories, Barcelona, Spain). Animals weighing 130 g were housed singly at 22 °C with a light/dark period of 12 h (lights on at 09:00 h) and with free access to food and water. After an adaptation period of 4 days, the hamsters were randomly distributed into two experimental groups (n = 16 per group) and fed either a normal-fat diet (NFD; D10051906) or a high-fat diet (HFD; D10051907) (Research Diets Inc., New Brunswick, NJ, USA) *ad libitum*. The composition of the diets is shown in Supplementary Table S1. The NFD provided 10% energy as fat, whereas the HFD provided 21% energy as fat because of the high content of lard. Eight animals of each group were sacrificed on day 15 (15d) and the other 8 on day 30 (30d).

A second study was conducted using a different strategy to induce dyslipidemia. For this purpose, 8-week-old male Golden Syrian hamsters (Janvier, Le Genest-St-Isle, France) weighing 110 g were housed individually in cages at 22 °C with a light/dark period of 12 h and free access to food and water. After an adaptation period of 2 weeks in which animals were fed an NFD, they were randomly assigned to six groups (n = 9–10 per group). Three groups continued the experiment for 4 days (4d) to detect early changes in the metabolome profile between groups, whereas the other three groups were sacrificed at 30d. The experimental design is described below. Two groups were maintained on the NFD and served as control animals (C-4d and C-30d groups), two other groups were fed the lard-based HFD (HFD-4d and HFD-30d groups) and the last two groups were fed the NFD and periodically injected with 50 mg of P407 per kg body weight every 72 h to induce dyslipidemia (P407-4d and P407-30d groups). Until now, P407 has been extensively used in mice^15, 26^ and rats^14, 27, 28^ but, to the best of our knowledge, only one study has been conducted in Golden Syrian hamsters that were periodically injected with P407 to induce dose-dependent hyperlipidemia^29^. In the present work, we have utilized a lower dose of P407 than the dose used by Liu *et al*.^29^. Based on preliminary studies, this quantity was selected as the adequate dose to achieve the development of moderate hypertriglyceridemia and hypercholesterolemia in hamsters (see Supplementary Fig. S1). The animals that were not treated with P407 received injections of vehicle (0.9% NaCl) with equal frequency. The hamsters were sacrificed 24 h after the last administration of P407 or vehicle. Lean and fat mass measurements (in grams) were performed without anesthesia on the first and the last days of the study using an EchoMRI-700™ (Echo Medical Systems, LLC., TX, USA).

In both experiments, the hamsters were deprived of food for 6 h on the day of sacrifice and euthanized under anesthesia (pentobarbital sodium, 80 mg per kg body weight). Blood was collected by cardiac puncture and serum was obtained by centrifugation (2,000 g for 15 min). The livers were dissected, weighted and immediately frozen in liquid nitrogen. All the samples were stored at −80 °C until further analyses.

### Serum measurements

Enzymatic colorimetric kits were used for the determination of glucose and triglycerides (QCA, Barcelona, Spain), phospholipids (Spinreact, Girona, Spain), non-esterified free fatty acids (NEFAs) (WAKO, Neuss, Germany), TC, HDLc and LDLc (Bioassay systems, Hayward, CA, USA). The circulating levels of the enzymes lecithin-cholesterol acyltransferase (LCAT), group IIA secreted phospholipase A_2_ (sPLA_2_-IIA), and paraoxonase-1 (PON1) were measured with hamster ELISA kits (MyBioSource, San Diego, CA, USA).

### Sample preparation for non-targeted metabolomics

Serum extracts were prepared using a procedure similar to the method described by Jové *et al*.^20^. Briefly, 90 µL of cold methanol containing phenylalanine^13^C as the IS (10 ppm) and BHT (1 µM) as an antioxidant were added to 30 µL of serum, vortexed for 1 minute and incubated at −20 °C for 1 h to precipitate proteins. Samples were centrifuged at 12,000 g for 5 min at 4 °C and the supernatants were collected and dried in a SpeedVac (Thermo Fisher Scientific, Waltham, MA, USA). Samples were re-suspended in 100 µL of Milli-Q water containing 0.2% acetic acid:methanol 0.2% acetic acid (1:1) prior to injection.

### LC-MS and LC-MS/MS analyses

Both untargeted and targeted analyses were performed on serum extracts using an UHPLC 1290 coupled to a Q-TOF 6550 mass spectrometer (Agilent Technologies, Palo Alto, CA, USA). The temperature in the autosampler was maintained at 4 °C throughout the analysis, and the samples were randomized.

Solvent A consisted of 0.2% acetic acid and solvent B consisted of methanol with 0.2% acetic acid. Two microliters of sample were applied to a Zorbax SB-Aq (1.8 µm particle size, 2.1 mm internal diameter × 50 mm length) analytical column maintained at 60 °C and equipped with a Zorbax SB-C8 (3.5 µm, 2.1 × 30 mm) guard column, also from Agilent Technologies. Chromatographic separation was performed by continuous gradient elution at flow rate of 0.6 mL/min starting at 2% B and increasing to 98% B in 13 min, where it was maintained isocratically for 6 min. The chromatographic system was returned to the initial conditions in 1 min, followed by a 5-min equilibration prior to the subsequent injection. Ionization in the mass spectrometer was performed by AJS ESI operated either in positive (+ESI) and negative (−ESI) mode with the following settings: nebulizer gas, nitrogen at a pressure of 45 psi; desolvation gas flow rate, 9 L/min at 325 °C; source temperature and gas flow rate, 150 °C and 12 L/min, respectively; capillary voltage, 4 kV; fragmentor, 125 V. Accurate LC-MS mass spectra were acquired over the 40–1600 *m/z* range at a scan rate of 1.5 spectra/s. A reference solution was used for continuous calibration with the following reference masses: 121.0509 and 922.0098 *m/z* for +ESI and 119.0363 and 980.0164 *m/z* for −ESI. Quality controls (QC) were prepared mixing 10 µL of each serum extract. At the start of the run, eight QC were injected for column conditioning and then every ten samples to assess instrument stability. For targeted analyses, the instrument was operating in MS and MS/MS modes, and the other analytical conditions were the same as described above. The MS/MS spectra of the metabolites were obtained at collision energies of 10 and 20 eV.

### Data processing and metabolite identification

All the software programs were provided by Agilent Technologies (Palo Alto, CA, USA). MassHunter Data Acquisition software was used to generate profile peak data from the spectra and MassHunter Qualitative Analysis software was used to obtain the molecular features of the samples. The peaks were aligned using Mass Profiler Professional (MPP) and mass and retention time (RT) windows of 0.1% ± 0.15 min and 15.0 ppm ± 2.0 mDa, respectively. Each chemical entity obtained from MPP is associated with a particular neutral mass, RT and abundance value. The list of molecular entities was filtered by selecting only those entities that were present in at least 80% of the samples in the same group. The normalized abundances were obtained by performing a base-2 logarithmic transformation of the ratio of each analyte to the IS. Candidate biomarkers were first identified using metabolite databases (METLIN^30^, HMDB^31^, and LIPID MAPS^32^). Finally, the structural information about the molecules of interest was provided by the fragmentation data obtained at different collision energies.

### Total RNA isolation and gene expression analysis

Livers used for mRNA analysis were homogenized and total RNA was extracted with TRIzol Reagent and purified on RNeasy Mini Kit spin columns (Qiagen, Barcelona, Spain) according to the manufacturer’s protocols. RNA yield was quantified on a Nanodrop 1000 Spectrophotometer (Thermo Scientific, Madrid, Spain) and tested for purity by measuring A260/280 ratio. Total RNA (0.5 µg; in a final volume of 20 µL) was reverse transcribed into complementary DNA (cDNA) using the High-Capacity cDNA Reverse Transcription Kit (Applied Biosystems, Madrid, Spain). cDNAs were subjected to quantitative real-time reverse transcriptase-polymerase chain reaction (qRT-PCR) amplification using SYBR Green PCR Master Mix (Bio-Rad, Barcelona, Spain). All reactions were performed in triplicate. When possible, primers were designed to span an exon-exon junction at the conserved domain of the gene of interest, avoiding the problem of the possible contamination by genomic DNA. Primer sequences for the target genes are listed in Supplementary Table S2 and were obtained from Biomers.net (Ulm, Germany). The relative mRNA expression levels were calculated using the 2^−ΔΔCt^ method^33^ with *β-actin* as the reference gene and were normalized to the C-4d group.

### Statistical analysis

The results are presented as the means ± SEM from the indicated number of hamsters. The assumption of normality was determined using the Shapiro-Wilk test, and the homoscedasticity between groups was assessed using Levene’s test. Once these conditions were verified, differences among groups were assessed using two-way ANOVA to evaluate the main effects of the time (duration of the experiment) and the pro-dyslipidemic intervention (HFD and P407) and their interaction. For the untargeted analysis, the p-values were calculated using the Benjamini-Hochberg correction. When any of the effects was statistically significant, one-way ANOVA was used to determine the differences among all the means at once. Tukey’s *post hoc* test was applied when the variances were similar and the Games-Howell test was applied if this assumption was not fulfilled. Student’s t-test was used for single statistical comparisons. A two-tailed value of p < 0.05 was considered statistically significant. All statistical analyses were performed with Statistical Package for Social Sciences (IBM SPSS Statistics, version 19.0).

## Results

### Identification of a class of lipids involved in dyslipidemia progression

**Figure 1:**
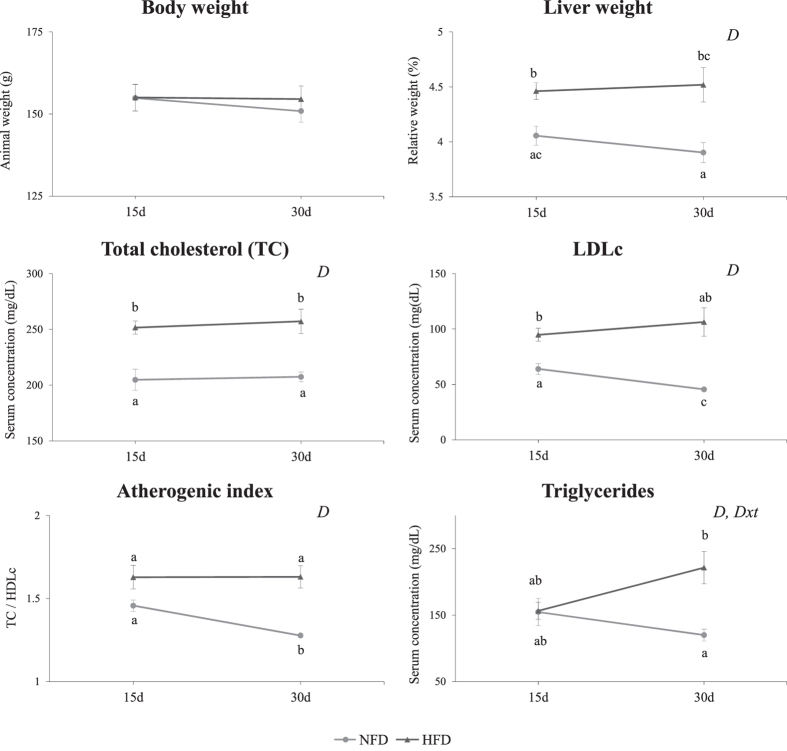
Biological characteristics of the hamsters from the initial study. Adult hamsters were distributed into two groups and fed either a normal-fat diet (NFD) or a high-fat diet (HFD). Half of the animals in each group were sacrificed on day 15 (15d) and the other half were sacrificed on day 30 (30d). Serum parameters were determined after a 6 h fast. The data are presented as means ± SEM (n = 8 per group). The statistical comparisons among groups were conducted using two- and one-way ANOVAs. *D*: the effect of the diet; *Dxt*: the interaction between diet and time (two-way ANOVA, p < 0.05). In each figure, different superscript lowercase letters (^a,b,c^) indicate significant different mean values (one-way ANOVA and Tukey’s or Games-Howell *post hoc* tests, p < 0.05). TC, total cholesterol; LDLc, low-density lipoprotein cholesterol; HDLc, high-density lipoprotein cholesterol.

Based on the results from the initial study, the development of dyslipidemia was successfully induced by the chronic administration of the HFD to the animals for 15d and 30d (Fig. 1). Hamsters fed the HFD showed higher amounts of TC, LDLc and triglycerides in serum, higher values for the atherogenic index (TC/HDLc) and greater relative liver weights than animals fed the NFD (p < 0.005, two-way ANOVA), without alterations in the body weight. However, time did not significantly affect any of these parameters, and only the circulating triglyceride levels were influenced by the interaction between diet and time (p = 0.008, two-way ANOVA).

Based on these findings, the state of dyslipidemia in hamsters fed the HFD for 15d was too similar to the hamsters fed for 30d. Nevertheless, the untargeted comparative analysis of the animal serum revealed that two lipid subclasses were differentially modified by HFD feeding according to the length of the treatment (Table 1). On the one hand, the circulating levels of five monoacylglycerophosphocholines, which are usually named lysophosphatidylcholines (Lyso-PCs), were increased in response to HFD intake (p < 0.050, two-way ANOVA), and the increase in all of the identified Lyso-PCs was restricted at 15d. On the other hand, although the diet had an overall effect on lysophosphatidylinositol (18:1) (Lyso-PI (18:1)) (p = 0.006, two-way ANOVA), the serum levels of all of the Lyso-PIs were at least residually increased in the HFD-fed animals compared to NFD-fed animals at 30d (p < 0.050, Student’s t-test), which was a later response than Lyso-PCs to HFD feeding. Other metabolites, including monoacylglycerols, amino acids and fatty acids such as arachidonic acid, were altered in the HFD-fed groups, but the glycerol-based lysophospholipids (Lyso-PLs) were the most representative lipid class. Concretely, this means that Lyso-PL category was the family of metabolites that exhibited the most numerous and deepest changes in the overall metabolome in this study. Furthermore, Lyso-PLs have been previously described as indicators of the health status in both animal and humans studies^34–36^. Therefore, taking in consideration all this information and evidence, this first study highlighted the importance of this family of compounds and their potential as suitable candidate biomarkers of dyslipidemia progression.

**Table 1:** Identification of the serum lysoglycerophospholipids (Lyso-PLs) that were significantly altered by the high-fat diet (HFD) compared to the normal-fat diet (NFD) after 15 and 30 days of feeding in the initial study.

Ionization mode	Ion *m/z* (MS)	Distinctive fragments (MS/MS)	Tentative identification^Ϯ^	Lipid subclass	Increased with HFD feeding
+ESI	[M + H]^+^: 524.3743	184.1, 104.1, 60.1	Lyso-PC (18:0)	Monoacylglycerophospho cholines	15d
	[M + H]^+^: 520.3440	184.1, 104.1, 60.1	Lyso-PC (18:2)		
	[M + H]^+^: 518.3251	184.1, 104.1, 60.1	Lyso-PC (18:3)		
	[M + H]^+^: 550.3871	184.1, 104.1, 60.1	Lyso-PC (20:1)		
	[M + H]^+^: 548.3719	184.1, 104.1, 60.1	Lyso-PC (20:2)		
−ESI	[M − H]^−^: 599.3218	419.2, 283.3, 79.0	Lyso-PI (18:0)	Monoacylglycerophospho inositols	30d
	[M − H]^−^: 597.3060	417.2, 281.8, 79.0	Lyso-PI (18:1)		
	[M − H]^−^: 621.3060	441.0, 305.1, 79.0	Lyso-PI (20:3)		
	[M − H]^−^: 619.2906	439.0, 303.2, 79.0	Lyso-PI (20:4)		

^Ϯ^According to the LIPID MAPS^32^ glycerophospholipid nomenclature, the structure of the acyl chain is indicated within parentheses in the ‘Headgroup (*sn1* or *sn2*)’ format. The headgroup is assumed to be attached to the *sn3* position of glycerol.

Abbreviations: Lyso-PC, lysophosphatidylcholine; Lyso-PI, lysophosphatidylinositol.

### Evaluation of the involvement of Lyso-PLs in the etiology and early progression of dyslipidemias

We performed a second animal study in which we induced an earlier stage of dyslipidemia by only feeding the animals the HFD for 4d to analyze the ability of this class of compounds to serve as biomarkers of dyslipidemia-related diseases. Furthermore, an additional strategy was used to induce hyperlipidemia that was not influenced by the diet in this second study. Therefore, two groups of animals were treated with P407 for 4d or 30d.

#### Health status of animals with two diverse forms of dyslipidemia

The biometric and serum analyses of the six groups of hamsters are reported in Table 2. At the start of the experiment, all the groups exhibited similar body weights and fat percentages. Total body weight was not affected by the treatments throughout the study, whereas the overall and relative liver weights of the HFD- and P407-treated groups increased, mainly in the animals submitted to the 30d treatments, in which significant differences were observed compared with the C-30d group. Nonetheless, only significant effects of the HFD on adiposity were observed, but not P407 administration. HFD-fed animals displayed numerically greater adiposity than the C groups at both times (Table 2). Although the *post hoc* analysis only revealed a significant difference in response to HFD intake at 4d, a residual increase was also noted in the HFD-30d animals compared to the corresponding C group (p = 0.014, Student’s t-test). Regarding the biochemical parameters, the pro-dyslipidemic intervention had an overall significant effect by increasing the circulating levels of TC and its main fractions. Although both treatments influenced the TC levels, the increase in the levels of the fraction corresponding to the evaluated lipoproteins is mainly due to HFD intake. According to the *post hoc* analysis, the TC and LDLc levels in the HFD-fed animals were altered throughout the study, but the response of HDLc was the slowest and increased after 30d of feeding. Conversely, the TC levels in the P407-treated animals increased rapidly but then stabilized and approached the levels of the C-30d group. The atherogenic index, which is calculated as the ratio of TC and HDLc, was also modulated by the pro-dyslipidemic intervention. More specifically, P407-treated animals had increased values of the index compared to each C group beginning at 4d, whereas significant alterations in response to HFD feeding were only observed at 30d. On the other hand, sharp increases in the serum triglyceride levels of approximately 200% and 150% were produced by P407 after 4d and 30d of treatment, respectively, whereas no significant changes were detected between the HFD-fed animals and C groups. The intervention also induced a significant increase in the serum phospholipid and glucose levels that was more pronounced for phospholipids than for glucose. The increase in the phospholipid levels was generally induced by both pro-dyslipidemic treatments, although the *post hoc* analysis only revealed a significant difference in the P407-treated animals at 4d. However, the overall effects on glucose were due to the diet, which showed an increasing trend, particularly at 30d (p = 0.016, Student’s t-test), but P407 did not induce alterations in the glucose levels. Finally, the serum NEFA levels were the only component that was not affected by either treatment throughout the study.

**Table 2:** Biometric and serum biochemical parameters measured in each group of hamsters from the second experiment.

	C-4d	HFD-4d	P407-4d	C-30d	HFD-30d	P407-30d	2-way ANOVA
**Biometric parameters**							
Initial body weight (g)	113.83 ± 2.27	111.66 ± 2.84	112.59 ± 3.36	113.94 ± 1.52	115.45 ± 1.83	112.53 ± 1.83	
Initial fat (%)	10.34 ± 0.74	10.39 ± 0.72	10.48 ± 1.15	11.70 ± 0.41	10.47 ± 0.61	11.02 ± 0.50	
Final body weight (g)	115.56 ± 1.97^a^	114.87 ± 2.80^a^	114.94 ± 3.48^a^	124.20 ± 0.96^ab^	129.60 ± 2.38^b^	125.80 ± 1.93^b^	*t*
Fat increase (%)	−0.37 ± 0.17^a^	0.70 ± 0.24^b^	−0.33 ± 0.17^a^	1.47 ± 0.43^c^	3.72 ± 0.67^c^	2.13 ± 0.35^c^	*I, t*
Liver weight (%)	3.47 ± 0.12^a^	3.65 ± 0.04^a^	3.79 ± 0.11^ab^	3.45 ± 0.10^a^	4.07 ± 0.09^b^	4.04 ± 0.08^b^	*I, t*
**Biochemical parameters**							
TC (mg/dL)	162.36 ± 6.10^a^	231.31 ± 12.16^bc^	231.41 ± 14.09^bc^	170.16 ± 8.00^a^	251.30 ± 8.91^b^	200.90 ± 8.28^ac^	*I, Ixt*
HDLc (mg/dL)	73.22 ± 2.64^a^	83.42 ± 3.60^ab^	77.08 ± 3.31^a^	73.70 ± 5.59^a^	94.09 ± 4.39^b^	73.80 ± 3.12^a^	*I*
LDLc (mg/dL)	53.94 ± 1.43^bc^	69.82 ± 4.25^a^	65.65 ± 3.84^ac^	47.76 ± 1.85^b^	67.92 ± 3.01^a^	46.92 ± 4.12^b^	*I, t, Ixt*
Atherogenic index (TC/HDLc)	2.34 ± 0.11^ac^	2.77 ± 0.09^ab^	3.02 ± 0.17^b^	2.25 ± 0.10^c^	2.69 ± 0.08^ab^	2.73 ± 0.07^ab^	*I*
Triglycerides (mg/dL)	149.60 ± 15.78^a^	187.16 ± 15.57^ac^	457.21 ± 58.76^bd^	219.82 ± 11.93^ce^	269.28 ± 16.38^de^	533.02 ± 50.43^b^	*I, t*
Phospholipids (mg/dL)	277.96 ± 11.82^a^	319.03 ± 11.53^ab^	356.52 ± 15.70^b^	288.21 ± 12.88^ac^	338.91 ± 12.50^ab^	347.34 ± 20.14^bc^	*I*
Glucose (mg/dL)	231.88 ± 14.99	253.67 ± 9.90	209.26 ± 12.59	200.97 ± 16.97	254.67 ± 10.88	220.71 ± 16.20	*I*
NEFAs (mM)	0.46 ± 0.09	0.36 ± 0.05	0.50 ± 0.06	0.55 ± 0.10	0.79 ± 0.16	0.77 ± 0.14	*t*

The hamsters were distributed into six groups, depending on the treatment and the duration of the experiment: control group-4 days (C-4d), diet-induced dyslipidemia-4 days (HFD-4d), pro-dyslipidemic agent-4 days (P407-4d), control group-30 days (C-30d), diet-induced dyslipidemia-30 days (HFD-30d), and pro-dyslipidemic agent-30 days (P407-30d). The diet-induced dyslipidemia was generated by a high-fat diet (HFD) providing 21% energy as fat and 0.92% as cholesterol. The pro-dyslipidemic agent was Poloxamer 407 (P407) and the dose was 50 mg/kg of body weight. P407 animals were fed the control diet and received intraperitoneal injections of the drug every 3 days. Non-P407 hamsters received injections of vehicle (0.9% NaCl) with equal periodicity. The animals were sacrificed 24 h after the last administration of P407 or vehicle and after a 6 h fast. The concentrations of serum parameters were determined at the end of the experiment. Relative fat and liver weights were determined using the formula 100* (tissue weight/body weight) and expressed as a percentage of the total body weight. Fat increase was calculated as the difference in the relative fat mass at each time point from the initial value for each animal. The data are presented as means ± SEM (n = 9–10). The statistical comparisons among groups were conducted using two- and one-way ANOVAs. A two-tailed p-value < 0.05 was considered statistically significant. For the two-way ANOVA: *I*: the effect of the pro-dyslipidemic intervention (HFD and P407); *t*: the effec*t* of time; *Ixt*: the interaction between the two factors. When one-way ANOVA was also significant, the *post hoc* analysis was used. In each row of the table, different superscript lowercase letters (^a,b,c^) indicate significant different mean values (Tukey’s or Games-Howell tests). TC, total cholesterol; HDLc, high-density lipoprotein cholesterol; LDLc, low-density lipoprotein cholesterol; NEFAs, non-esterified free fatty acids.

**Figure 2:**
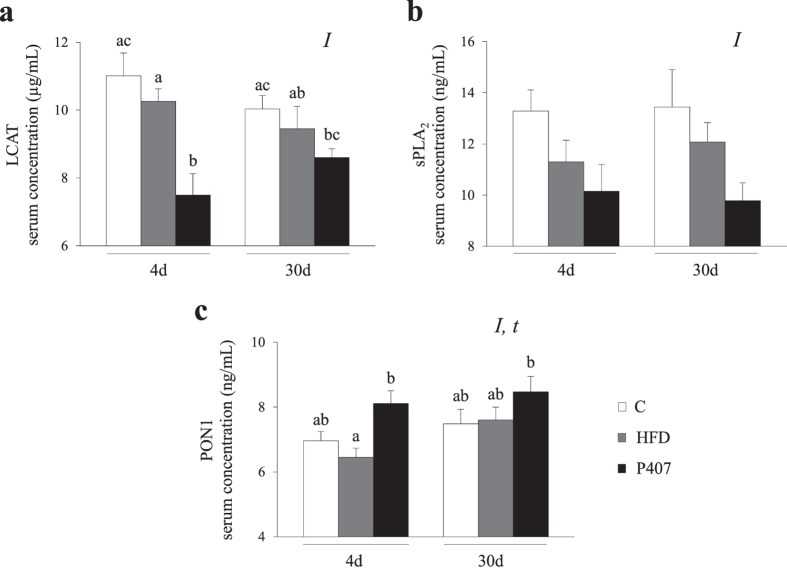
Circulating levels of different enzymes involved in Lyso-PL synthesis in hamsters with different degrees of dyslipidemia. The animals were distributed into three groups: C, group fed a normal-fat diet (NFD); HFD, group fed a high-fat diet; and P407, group fed an NFD and periodically injected with the pro-dyslipidemic agent P407. Half of the animals in each group were sacrificed on day 4 (4d) and the other half were sacrificed on day 30 (30d). The dose of P407 was 50 mg/kg every 3 days. Non-P407 hamsters received injections of vehicle (0.9% NaCl) with equal periodicity. All animals were sacrificed 24 h after the last administration of P407 or vehicle and after a 6 h fast. The concentrations of serum parameters were determined at the end of the experiment. The data are presented as means ± SEM (n = 9–10). The statistical comparisons among groups were conducted using two- and one-way ANOVAs. *I:* the effect of the pro-dyslipidemic intervention (HFD and P407); *t:* the effect of time (two-way ANOVA, p < 0.05). In each figure, different superscript lowercase letters (^a,b,c^) indicate significant different mean values (one-way ANOVA and Tukey’s or Games-Howell *post hoc* tests, p < 0.05).

In addition, some of the enzymes involved in the extracellular release of Lyso-PLs were evaluated in these hamsters. As shown in Fig. 2, we investigated the serum levels of the enzymes LCAT, sPLA_2_-IIA and PON1. Overall, both treatments induced a decrease in the serum LCAT levels (p < 0.001, two-way ANOVA), which was much more evident in the groups treated with P407 for 4d and 30d compared with their respective control counterparts (32% and 14% decreases at 4d and 30d, respectively) (Fig. 2a). As shown in Fig. 2b, the serum sPLA_2_-IIA concentrations exhibited similar responses to both HFD intake and the P407 treatment (p = 0.003, two-way ANOVA) as the LCAT levels, with a more marked decline in the P407-treated animals than in HFD-fed groups compared with the controls (24% and 27% decreases at 4d and 30d, respectively; p = 0.032 and p = 0.049, Student’s t-test). Although the two-way ANOVA revealed overall effects of the pro-dyslipidemic intervention (p = 0.005) and time (p = 0.036) on the circulating levels of PON1, the pair-wise comparisons did not reveal significant differences at any time when the HFD-fed and the P407-treated animals were compared with the C groups (Fig. 2c). However, the P407-treated animals displayed numerically greater serum PON1 levels than C groups, mainly at 4d (p = 0.031; Student’s t-test).

Based on these results, the P407 treatment induced greater changes in the levels of enzymes involved in the release of Lyso-PLs into the bloodstream than HFD intake.

#### Relative expression of hepatic genes implicated in the extracellular release of Lyso-PLs

**Figure 3:**
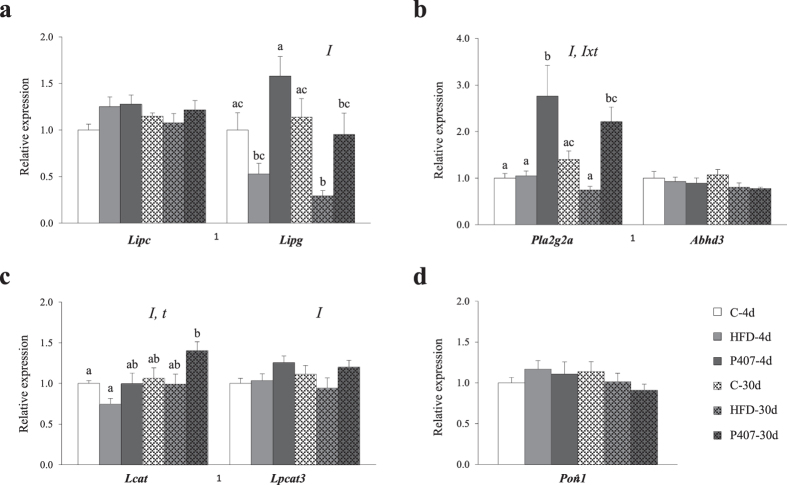
Relative expression levels of hepatic genes implicated in the regulation of the circulating Lyso-PL levels. (**a**) Lipases: *Lipc*, hepatic lipase; *Lipg*, endothelial lipase. (**b**) Phospholipases: *Pla2g2a*, phospholipase A_2_ group IIA; *Abhd3*, abhydrolase domain-containing 3. (**c**) Acyltransferases: *Lcat*, lecithin-cholesterol acyltransferase; *Lpcat3*, lysophosphatidylcholine acyltransferase 3. (**d**) *Pon1*, Paraoxonase 1. The hamsters were assigned to six groups, depending on the pro-dyslipidemic treatment and duration of the experiment: control group-4 days (C-4d), high-fat diet-4 days (HFD-4d), pro-dyslipidemic agent-4 days (P407-4d), control group-30 days (C-30d), high-fat diet-30 days (HFD-30d), and pro-dyslipidemic agent-30 days (P407-30d). Total RNA was isolated from the liver and subjected to qRT-PCR analysis. The relative expression levels were determined using *β-actin* as the reference gene and were normalized to the C-4d group. The data are presented as means ± SEM (n = 9–10). The statistical comparisons among groups were conducted using two- and one-way ANOVAs. *I:* the effect of the intervention (HFD and P407); *t:* the effect of time; *Ixt*: the interaction between the two factors (two-way ANOVA, p < 0.05). For each gene, different superscript lowercase letters (^a,b,c^) indicate significant different mean values (one-way ANOVA and Games-Howell *post hoc* test, p < 0.05).

Impaired hepatocyte functions are responsible for alterations in endogenous plasma lipid and lipoprotein levels and the subsequent development of dyslipidemias^37^. For this reason, we evaluated the relative expression levels of some hepatic genes related to the regulation of extracellular Lyso-PL levels (Fig. 3). The pro-dyslipidemic intervention had significant effects on the relative expression levels of the genes encoding the lipase *Lipg*, the phospholipase *Pla2g2a* (p < 0.001) and the acyltransferases *Lcat* (p = 0.010) and *Lpcat3* (p = 0.033) (two-way ANOVA). Specifically, the pair-wise comparisons revealed that the mRNA levels of *Pla2g2a* were significantly higher in P407-4d animals than the levels in the C-4d group, and very similar trends were observed for *Lipc*, *Lipg* and *Lpcat3*, showing that the P407-treated animals expressed higher levels of these genes than the C group at 4d (p < 0.050, Student’s t-test). Furthermore, after 30d of pharmacological treatment, the P407-30d hamsters displayed a residual increase in *Lcat* expression and decrease in *Abhd3* mRNA levels compared with the C-30d group (p < 0.050, Student’s t-test). Moreover, HFD feeding induced alterations in the expression of genes related to hepatic Lyso-PL metabolism, but to a lesser extent than the P407 treatment and, generally, with opposite trends. Thus, compared to the corresponding C group at each time, the expression of *Lcat* and *Lipc* was decreased and increased, respectively, in the HFD-4d group (p = 0.005 and p = 0.049, Student’s t-test), whereas *Pla2g2a* expression decreased in the HFD-30d hamsters (p = 0.002, Student’s t-test). In addition, the levels of the *Lipg* mRNA were decreased in the HFD-fed animals compared with the control hamsters at both times (p = 0.024 and p = 0.001, Student’s t-test). No significant differences in the expression of any evaluated gene were observed between the C groups.

#### Non-targeted metabolomics of Lyso-PLs and metabolite identification

After confirming that the two pro-dyslipidemic treatments studied here utilize different mechanisms in the liver, we analyzed the circulating metabolome using a non-targeted approach to determine the whole range of altered Lyso-PLs and confirm their suitability as biomarkers of progression and dyslipidemia typology.

After aligning and filtering the molecular entities identified in the six groups of hamsters, the results of the LC-MS/MS analysis showed two characteristic fragmentation patterns that exhibited changes in the dyslipidemic groups (Table 3). Based on the exhaustive study of the fragmentation data, we concluded that both patterns represented Lyso-PL species. In addition, all these Lyso-PLs identified were molecular species containing the acyl chain in *sn1*-position, which generally is the most common circulating isomer for these metabolites^38^. When collision energies of 10 V were applied in the positive ionization mode, several daughter ions were generated: a) the loss of the phosphorylethanolamine head group ([M + H]^+^ −141), b) the loss of water ([M + H]^+^ −18), c) the ion corresponding to the ethanolamine ([M + H]^+^  = 62) and its fragment, d) the ethylamine ([M]^+^  = 44), e) the phosphocholine ([M – H + 2 H]^+^  = 184), and f) choline ions ([M + H]^+^  = 104). The daughter ions described in *a-d* are from lysophosphatidylethanolamine (Lyso-PE) species, whereas the daughter ions described in *e-f* are fragments from the Lyso-PCs identified in the previous study. Published spectra for these lipid subclasses are available in the METLIN database^30^ and Liebisch *et al*.^39^. The RTs of Lyso-PLs should also be examined. Lyso-PLs with identical acyl chain lengths but different double bond numbers had consistent behaviors. A greater number of double bonds reduced the retention of the particles on the chromatographic column, corresponding to lower RTs.

**Table 3:** LC-+ESI-MS/MS identification of the altered Lyso-PLs in the serum of hamsters from the second study.

Neutral mass	RT (min)	Collision energy		Formula (score)^¥^	Lipid subclass	Tentative identification^Ϯ^
		10 V	20 V			
475.2661	10.85	335.3, 44.1, 458.3	44.1, 335.3	C_23_H_42_NO_7_P (86.26)	Monoacylglycerophospho ethanolamines	Lyso-PE (18:3)
451.2679	10.92	311.3, 44.1, 434.3	44.1, 311.3	C_21_H_42_NO_7_P (93.69)		Lyso-PE (16:1)
477.2854	11.20	337.3, 44.1, 62.1, 460.3	44.1, 337.3	C_23_H_44_NO_7_P (81.06)		Lyso-PE (18:2)
501.2853	11.21	361.3, 44.1, 62.1, 484.3	44.1, 361.3	C_25_H_44_NO_7_P (91.87)		Lyso-PE (20:4)
525.2847	11.23	385.3, 44.1, 62.1, 508.3	44.1, 385.3	C_27_H_44_NO_7_P (94.58)		Lyso-PE (22:6)
453.2852	11.41	313.3, 44.1, 62.1, 436.3	44.1, 313.3	C_21_H_44_NO_7_P (63.07)		Lyso-PE (16:0)
547.3634	11.93	184.1, 104.1	184.1, 104.1, 60.1	C_28_H_54_NO_7_P (98.74)	Monoacylglycerophospho cholines	Lyso-PC (20:2)
549.3767	12.27	184.1, 104.1	184.1, 104.1, 60.1	C_28_H_56_NO_7_P (64.24)		Lyso-PC(20:1)

^¥^The molecular formula score was calculated using the ‘Molecular Formula Generator’ algorithm included in the MassHunter Qual software.

^Ϯ^According to the LIPID MAPS^32^ glycerophospholipid nomenclature, the structure of the acyl chain is indicated within parentheses in the ‘Headgroup (*sn1* or *sn2*)’ format. The headgroup is assumed to be attached at the *sn3* position of glycerol.

Abbreviations: RT, retention time; Lyso-PE, lysophosphatidylethanolamine; Lyso-PC, lysophosphatidylcholine.

**Figure 4:**
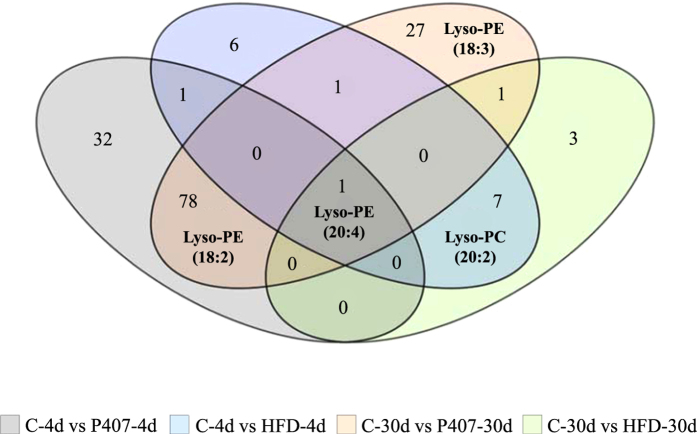
Venn diagram displaying the implications of the identified circulating lyso forms in the four comparisons of interest. The statistical comparisons between the six groups (n = 9–10) were assessed using two- and one-way ANOVAs. The cut-off point for significance was p < 0.05 and for the two-way ANOVA, the p-value of the ‘intervention’ effect was calculated using the Benjamini-Hochberg correction. The figures indicate the number of significant metabolites for each comparison of interest, which were determined using Tukey’s *post hoc* test. Previously, the metabolites that exhibited significant changes among control groups (C-4d vs C-30d) were discarded. The areas where the ellipses overlap show the significant metabolites, particularly the Lyso-PLs, shared by the specific comparisons. Lyso-PE (20:4) was the only metabolite whose abundance was altered by both diet and the pro-dyslipidemic agent in the short- and long-term assessments.

According to the two-way ANOVA, six Lyso-PEs and two Lyso-PCs exhibited significant changes in the serum of hamsters subjected to HFD feeding and/or P407 treatment for 4d or 30d (Table 4). However, the *post hoc* analysis performed on the six groups revealed a different trend that depended on the Lyso-PL class: Lyso-PC levels - particularly the Lyso-PC (20:2) levels - were increased in the diet-induced dyslipidemic groups, whereas Lyso-PE abundances mainly increased in the P407-treated groups. Moreover, Lyso-PEs allow us to distinguish the groups with P407-induced dyslipidemia. At 4d, the serum Lyso-PE (18:2) and (20:4) levels were already increased, but the (18:3) and (22:6) levels changed over a longer period. Interestingly, as shown in Table 4 and in Fig. 4, Lyso-PE (20:4) was the unique metabolite that showed a significant increase in response to both the HFD and P407 treatments at 4d and at 30d; in addition, its levels remained constant between both C groups. Therefore, at first glance, Lyso-PE (20:4) may be a putative novel biomarker of dyslipidemia.

**Table 4:** Normalized serum Lyso-PL levels that were significantly altered in the dyslipidemic groups.

Compound name		C-4d	HFD-4d	P407-4d	C-30d	HFD-30d	P407-30d	2-way ANOVA (*I*)	
								*p*	*p (Corr)*
Lyso-PE	(20:4)	−0.33 ± 0.05^a^	0.04 ± 0.06^bc^	0.12 ± 0.11^bc^	−0.18 ± 0.08^ac^	0.18 ± 0.08^b^	0.32 ± 0.10^b^	<0.001	<0.001
	(18:2)	−0.17 ± 0.08^a^	−0.06 ± 0.03^ac^	0.28 ± 0.12^bc^	−0.19 ± 0.10^a^	−0.02 ± 0.09^ac^	0.43 ± 0.14^b^	<0.001	<0.001
	(18:3)	−0.10 ± 0.18^a^	−0.22 ± 0.09^a^	0.36 ± 0.17^ab^	−0.23 ± 0.19^a^	−0.30 ± 0.14^a^	0.72 ± 0.19^b^	<0.001	<0.001
	(22:6)	−0.43 ± 0.10^a^	−0.08 ± 0.10^ab^	0.05 ± 0.17^ab^	0.10 ± 0.11^b^	−0.01 ± 0.08^ab^	0.81 ± 0.11^c^	<0.001	<0.001
	(16:1)	−0.26 ± 0.26^a^	−0.25 ± 0.13^a^	0.13 ± 0.24^ab^	0.23 ± 0.17^ab^	−0.20 ± 0.14^a^	0.95 ± 0.21^b^	0.001	0.02
	(16:0)	−0.14 ± 0.11^a^	−0.13 ± 0.09^a^	0.00 ± 0.21^ab^	0.06 ± 0.17^ab^	−0.34 ± 0.20^a^	0.67 ± 0.18^b^	0.005	0.09
Lyso-PC	(20:2)	−0.60 ± 0.17^a^	0.24 ± 0.10^bc^	−0.58 ± 0.22^a^	−0.09 ± 0.19^ac^	0.75 0.19^b^	−0.16 ± 0.21^ac^	<0.001	<0.001
	(20:1)	−0.08 ± 0.16	0.35 ± 0.10	−0.37 ± 0.22	−0.30 ± 0.12	0.27 ± 0.24	−0.29 ± 0.21	0.003	0.08

The levels of each metabolite were transformed to a base-2 logarithm, normalized to the internal standard and to the mean levels of the C-4d samples. The data are presented as the means of the normalized abundances ± SEM (n = 9–10). The two-way ANOVA cut-off point was calculated using the Benjamini-Hochberg correction to avoid false positives. The p-values for the main factor ‘intervention’ without (*p*) and with correction (*p(Corr)*) are shown. *I*: the effect of the pro-dyslipidemic intervention (HFD and P407 administration). When one-way ANOVA was also significant (p < 0.05), the *post hoc* analysis was used. In each row of the table, different superscript lowercase letters (^a,b,c^) indicate significant different mean values (Tukey’s test, p < 0.05).

## Discussion

Chronic administration of the HFD progressively induced dyslipidemia in adult hamsters after 4, 15 and 30 days of feeding. The biological parameters that first responded to the dietary treatment were the circulating TC and LDLc levels and adiposity index because these parameters increased significantly after 4d of the intervention; at 15d, the consequences of the metabolic disorder started to be observed in the liver weights of the dyslipidemic animals. Finally, the atherogenic index increased after 30d of treatment compared to the controls. These findings are compatible with other studies conducted in HFD-fed hamsters^20, 40–42^. Consistent with the main objective of the current study, we introduced an additional group of hamsters in which dyslipidemia was induced through periodic injections of P407 instead of by the diet. Despite the low dose used, P407 induced a type of dyslipidemia characterized by a rapid and dramatic increase in the serum triglyceride levels throughout the study, in addition to early hypercholesterolemia (Table 2). The dose-dependent response to P407 was considerably greater for triglycerides than for TC^17^. Nevertheless, the P407-induced hyperlipidemia addressed in the present study was much milder than the form that has been reported in rodents^26, 28, 29, 43^ and did not exceed the cholesterol levels of the HFD-fed rodents^44, 45^. Furthermore, the adiposity index and serum levels of HDLc and LDLc were not affected by the chemical treatment, whereas the atherogenic index was altered more quickly than in animals administered the HFD, as an increase was already observed at 4d. Thus, hamsters that have been periodically treated with P407 are also a good model for studying atherogenic dyslipidemia^18, 26, 29^. Interestingly, the liver weights of the animals increased after 30d in the groups treated with both diet and chemical treatments.

Lyso-PLs were the main metabolites with altered circulating levels that were identified in the dyslipidemia-induced hamsters. These biologically active molecules are involved in a broad range of physiological and pathological processes, such as inflammation, apoptosis, reproduction, immunity, carcinogenesis, angiogenesis and regulation of metabolic diseases^46^. Some of these molecules have been proposed as biomarkers of diabetes^47^, the progression of atherosclerosis^48, 49^, obesity^50–52^ and different types of cancer^36, 53, 54^ in humans. In addition, *in vitro* studies using HepG2 cells have shown differences in the patterns of secreted Lyso-PLs under palmitate-induced, noncytotoxic, steatotic conditions^51^. This result is consistent with the serum levels and hepatic expressions of the Lyso-PL-related enzymes in our study, many of which were modulated by the pro-dyslipidemic intervention (Figs 2 and 3).

Circulating LCAT is exclusively synthetized by the liver and is an important enzyme involved in the reverse transport of cholesterol and maturation of HDL particles^55^. LCAT fulfils its function by transferring fatty acids from glycerophospholipids to cholesterol, contributing to the removal of cholesterol from the circulation and the release of Lyso-PLs^55^. In this study, P407 administration produced 30% and 15% decreases in the circulating LCAT levels after 4d and 30d of treatment, respectively (Fig. 2a); however, hepatic LCAT expression was only increased at the longer period (Fig. 3c). The increase in the plasma Lyso-PL levels seems to induce the drastic decrease in the LCAT levels after 4d of treatment, and the liver attempts to compensate for this shortage by increasing its expression. Moreover, increases in the biological activity of LCAT have been reported in P407-treated rodents^17, 28^ as a compensatory mechanism for the high plasma cholesterol loading in these animals. Modifications in exogenous LCAT activity and hepatic mRNA abundance have also been described in hamsters fed different dietary fats^42^, but our results did not show a significant effect on LCAT expression in the diet-induced dyslipidemic animals.

Lipases also contribute to cholesterol homeostasis due to their ability to hydrolyze triglycerides, cholesteryl esters and phospholipids within circulating lipoproteins, mediating important processes involved in the remodeling of these heterogeneous particles^55^. Endothelial lipase (EL) predominantly exerts phospholipase activity and prefers phosphatidylethanolamine as a substrate^46, 56, 57^. The hydrolysis of this favored substrate leads to the synthesis of Lyso-PEs, which constitute the most representative lipid subclass of our second study. Through its phospholipase activity on HDLc, EL enhances cholesterol removal from peripheral tissues and promotes hepatic accumulation^57^. In this study, both pro-dyslipidemic treatments (p < 0.001, two-way ANOVA) modified the hepatic expression of EL (*Lipg* locus) (Fig. 3a). After 4d, EL expression increased in the P407-treated group and decreased in the HFD-fed hamsters compared to the C-4d group. However, EL expression was also equally influenced by the time and the interaction (p = 0.062, two-way ANOVA), and the levels in both treated groups were decreased by half at day 30 compared to the earlier time points. The inactivation of lipases by P407 has been shown in different rodent models^28, 58^ and is related to liver injury^18^. Animal and human studies have revealed that lipases are negative regulators of HDLc levels^59, 60^. Moreover, HFD-fed animals showed the lowest EL expression levels in the liver and the highest plasma HDLc levels (Table 2).

sPLA_2_-IIA is a well-known pro-inflammatory enzyme that is involved in arachidonic acid release and eicosanoid production in the secretory compartment^61^. Interestingly, similar to EL, sPLA_2_-IIA has strong activity towards anionic phospholipids, such as phosphatidylethanolamines^61^. Some studies have described a sPLA_2_-mediated progression of liver impairments in rodents when the inducer was a chemical agent^62, 63^. Compared to the corresponding controls, we observed a substantial 200% increase in hepatic sPLA_2_-IIA expression (*Pla2g2a* locus) in the P407-4d animals and 60% increase (p = 0.043, Student’s t-test) in the P407-30d group (Fig. 3b), but not in the animals fed the HFD. This behavior is related to the substantial increase (p = 0.024, Student’s t-test) in EL expression in the P407-4d hamsters compared to the HFD-fed group (Fig. 3a) because EL has been positively associated with inflammation in rodents and humans^57, 64^. The decreased circulating sPLA_2_-IIA levels in the P407 groups might be explained by the observation that upon secretion by hepatocytes, the enzyme is rarely into the circulation due to the high rate of synthesis of Lyso-PLs. Based on these findings, animals that have been periodically treated with P407 develop dyslipidemia accompanied by hepatic inflammation, in contrast to the HFD-fed animals.

PON1 is an antioxidant enzyme of primarily hepatic origin that degrades oxidized cholesteryl esters and phospholipids within the lipoproteins^65, 66^ and has been proposed as a suitable non-invasive biomarker for human liver diseases^67^. Liver injury is usually associated with decreased serum activity and hepatic expression but increased circulating PON1 levels^68, 69^. In this study, the pair-wise comparisons did not reveal significant differences between the dyslipidemic and control hamsters, but the highest circulating levels were observed in the animals treated with the drug (Fig. 2c) and the largest decrease in hepatic expression of 20% was observed in the P407-30d group (Fig. 3d). The relation between P407 and PON1 has been described in injected mice, but our results in hamsters were not as clear^70^.

LPCAT3 is responsible for Lyso-PL reacylation and participates in the remodeling of phospholipids and membrane fluidity^71^. The composition of the cell membranes influences the lipid contents in the blood^72^. Mice lacking *Lpcat3* in the liver had lower circulating levels of lipids containing arachidonic acid and inefficiently mobilized triglycerides into very-low density lipoproteins (VLDL), resulting in lipid accumulation in hepatocytes^73^. The P407-treated groups, which were the animals with the highest serum Lyso-PE (20:4) levels, display an early tendency to overexpress *Lpcat3* (Fig. 3c), suggesting that hepatic VLDL production may be increased in these animals. In fact, although other authors have reported a shift in the distribution of cholesterol to the VLDL fraction as consequence of P407 administration^44^, we observed an increase in the TC levels in P407-4d hamsters, which did not correspond to either HDLc or LDLc (Table 2).

Thus, we have verified that dyslipidemia was induced by different hepatic mechanisms, depending on the etiology of the disorder^16, 27, 44, 74^. The dietary intervention seems to induce dyslipidemia by inactivating the hepatic genes involved in lipolysis, which induces the liver to sequester lipids^27, 45, 74–77^; however, oxidative stress and inflammation processes predominate in the animals treated with the chemical agent^17, 62, 64, 70^. Moreover, the development of hepatic steatosis without major inflammation has been described in rodents fed an HFD^74, 78^, whereas a histological examination of livers from P407-treated animals shows defenestration of the sinusoidal endothelium and the accumulation of large foamy Kupffer cells^10, 43^. These hepatic alterations are accompanied by the absence of steatosis and are also observed in cirrhosis^79^ and aging^80^. In this context, the Lyso-PLs levels may be used as a biomarker to distinguish typologies of hepatic disorders.

The non-targeted metabolomics evaluation presented in the first study suggested that this class of circulating lipids changes in accordance with the progression of the lipid disorder. The second study seems to confirm their suitability for the evaluation of the dyslipidemia state, showing a joint association of the Lyso-PEs levels with the progression of the disorder induced by the pharmacological treatment; however, the Lyso-PCs appear as specific biomarkers for the dyslipidemia from dietary origin. Among all of the Lyso-PLs, Fig. 4 shows the best putative candidate circulating biomarkers and discards those metabolites that were modified over time in the untreated animals. For their possible application in diagnosis, Lyso-PC (20:2), Lyso-PE (18:2) and Lyso-PE (20:4) were the Lyso-PLs that exhibited the earliest alterations in response to the dyslipidemia. Furthermore, Lyso-PE (20:4) was the unique metabolite that was modulated by both treatments throughout the study.

Due to the key role of lipids in the development and stability of atherosclerotic plaques, dyslipidemia is considerate a primary risk factor for atherosclerosis, which can remain asymptomatic for years. As described above, both types of treatments induced significant increases in the atherogenic index of the hamsters. Interestingly, other authors reported elevated amounts of several Lyso-PCs in atherosclerotic human aorta^81^ and within symptomatic versus asymptomatic carotid plaques^48^. On the other hand, increases in the plasma Lyso-PE (20:4) and (22:6) levels were associated with the occurrence of stable lesions but not unstable plaques^49^.

The elucidation of Lyso-PLs as early biomarkers of pathology is also consistent with recent studies indicating that chronic intake of an HFD dysregulates the plasma and hepatic levels of Lyso-PCs and Lyso-PEs in obese^82^ and non-obese^83^ mice. Consistent with our results, these authors observed altered circulating levels of Lyso-PCs (18:0), (18:2), (18:3) and (20:1) and Lyso-PEs (18:2), (20:4) and (22:6) in HFD-fed rodents. Actually, regarding hepatic disorders, some studies have examined hepatocarcinogenesis progression^36^ and selected certain members of Lyso-PEs as early biomarkers of pathology and altered patterns of Lyso-PCs as plasma indicators to distinguish the most advanced stage, namely the carcinoma phase. Moreover, increases in the serum Lyso-PEs (18:2) and (20:4) levels associated with early liver injury are reversed by fenofibrate therapy, an anti-dyslipidemic agent^76, 84^. Therefore, modifications in circulating Lyso-PE and Lyso-PC levels may be related to hepatic disorders and Lyso-PL patterns change with the progression of dyslipidemia-related diseases. Among all the species of Lyso-PLs, Lyso-PE (20:4) seems to possess specific potential as biomarker of the risk of developing lipid pathology.

We concluded that Lyso-PL evaluation could assist with the exhaustive diagnosis and management of lipid disorders since they provide information about the etiology of the dyslipidemia and, furthermore, allow researchers to determine the extent of dyslipidemia. In any case, the omics-based approach used in this study only provided a first approximation of this interesting issue. For instance, it would be necessary to perform a quantitative analysis of the lysophospholipidome by means of the use of an analytical method intended to Lyso-PLs including proper internal standards for these molecules, such as Lyso-PCs (13:0) or (19:0). Despite the abundant evidence in the literature on this topic, we believe that the future studies should be focused on the exhaustive evaluation of the whole family of Lyso-PLs in these animal models. Subsequently, extensive validation in subjects with dyslipidemia-related diseases is required to verify the suitability of the non-invasive biomarkers for use in the population.

## Electronic supplementary material

Supplementary information
