# Supplementary material for: Serum lysophospholipid levels are altered in dyslipidemic hamsters
Source: Sci Rep. 2017 Sep 5;7:10431. doi: 10.1038/s41598-017-10651-0 (PMC5585394; doi:10.1038/s41598-017-10651-0)
Supplement: Supplementary file 1 — Supplementary information [file 41598_2017_10651_MOESM1_ESM.pdf]

Serum lysophospholipid levels are altered in dyslipidemic hamsters

Susana Suárez-García[1], Antoni Caimari[2], Josep Maria del Bas[2]; Manuel Suárez[1*], and Lluís Arola[1,2]

Supplementary Information

	NFD	HFD
**Macronutrients**		
Protein (g/kg)	22	24
Carbohydrate (g/kg)	65	57
Fat		
g/kg	4	10
%	10	21
**Energy (kcal/g)**	4	4
**Ingredients (g/kg)**		
Casein, 80 mesh	220	220
L-Cysteine	3	3
Wheat starch	386.5	281
Maltodextrin 10	100	100
Dextrose	50	50
Sucrose	100	100
Cellulose, BW200	50	50
Coconut oil, 76	7.74	5
Flaxseed oil	5.16	4
Sunflower oil	30.1	10
Lard	0	71
Mineral mix S10022G	35	35
Vitamin mix V10037	10	10
Choline bitartrate	2.5	2.5
Cholesterol	0.03	0.92
**Fatty acids**		
SFA		
g/kg	10.9	28.6
%	26.5	33.7
MUFA		
g/kg	8.2	28.0
%	19.9	33.1
PUFA		
g/kg	22.1	28.2
%	53.6	33.2

Table S1. Composition of the diets. NFD, normal-fat diet; HFD, high-fat diet.

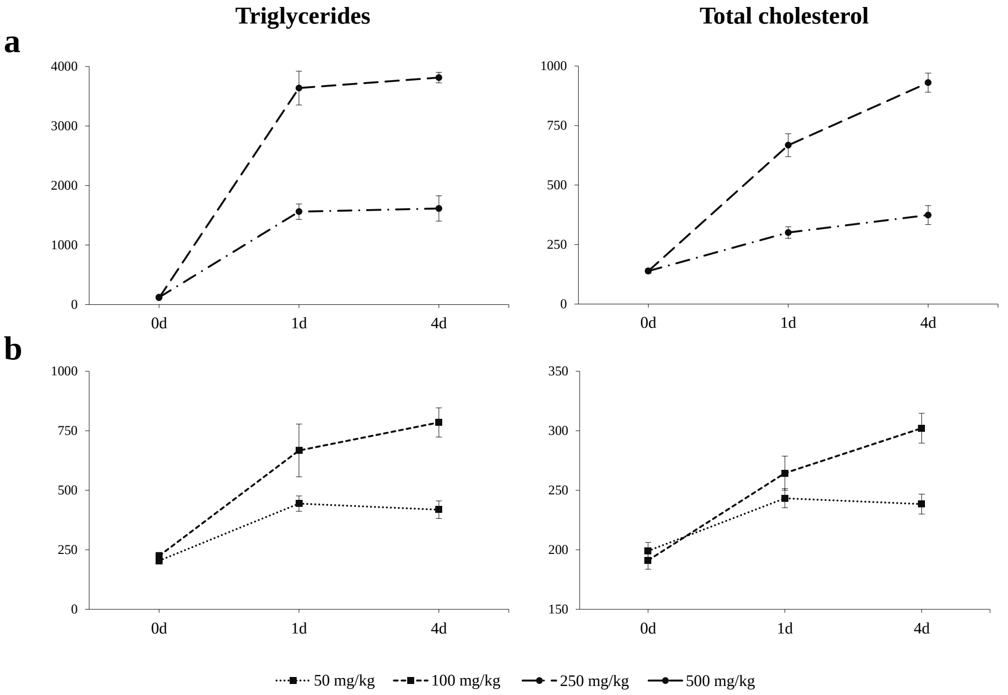

Figure S1. Determination of the adequate dose of P407 required to induce moderate hyperlipidemia in hamsters. In preliminary studies, animals were assigned to two groups (n = 7 per group) and treated with different doses of P407. These hamsters were fed a normal-fat diet and received two intraperitoneal injections of the drug 3 days apart (days 0 and 3) to induce hypertriglyceridemia and hypercholesterolemia. Blood was collected under fasting conditions 6 h before the first administration of P407 (0d) and 24 h after each injection, on days 1 (1d) and 4 (4d). (**a**) As a first approximation, we tested two concentrations similar to those commonly used in rodents (250 and 500 mg/kg), and both resulted in exaggerated hyperlipidemia. (**b**) In a second study, two lower doses were evaluated (50 and 100 mg/kg), and we selected 50 mg/kg as the most suitable dose of P407 among the doses assessed that induced stable and moderate dyslipidemia in adult hamsters.

Gene	Forward primer (5'-3')	Reverse primer (5'-3')
Lipc	GTGGTCGGGCTCAGAAAGTG	GTATGCCAGGGAGACCCAAT
Lipg	CCAAGGACCCAGAACACGAA	GCCACTCATCGTCCATCCAT
Pla2g2a	TGGCCCAATCCAGATCCAAG	AGTCGGTCGTAGCAACAGTC
Abhd3	GCTGTGCCTAAATGCTGTGG	TGGCCGCCGTAAGAAGTAAG
Pon1	GGGACTGCTGTTGGCATTCT	AGTCCAGTGCTGAGGAAAGC
Lcat	ACACTGGTGCAGAATCTGGTTA	ACTGTCTCATCCCGCACATAC
Lpcat3	TATGACAGCCGCCCTTTCTG	TCCTTCTGTGACCAGCCAAC
Actb	ACGTCGACATCCGCAAAGACCTC	TGATCTCCTTCTGCATCCGGTCA

Table S2. Hamster-specific primer sequences used for the gene expression analysis. *Lipc*, hepatic lipase; *Lipg,* endothelial lipase; *Pla2g2a*, phospholipase A_2 group IIA; *Abhd3,* abhydrolase domain-containing 3; *Pon1*, Paraoxonase 1; *Lcat,* lecithin-cholesterol acyltransferase; *Lpcat3,* lysophosphatidylcholine acyltransferase 3; *Actb*, beta-actin (housekeeping gene).
